# The relationship between vascular endothelial growth factor (VEGF) and amnestic mild cognitive impairment among older adults living with HIV

**DOI:** 10.1007/s13365-021-01001-y

**Published:** 2021-11-04

**Authors:** Vanessa B. Serrano, Jessica L. Montoya, Laura M. Campbell, Erin E. Sundermann, Jennifer Iudicello, Scott Letendre, Robert K. Heaton, David J. Moore

**Affiliations:** 1 Joint Doctoral Program in Clinical Psychology, San Diego State University, University of California, San Diego, La Jolla, CA, USA; 2 Department of Psychiatry, University of California, San Diego, La Jolla, CA, USA; 3 Department of Medicine, University of California, San Diego, La Jolla, CA, USA

**Keywords:** Aging, Cognition, HIV/AIDS, VEGF, Amnestic mild cognitive impairment

## Abstract

Older people with HIV (PWH) experience increased risk of age-related neurodegenerative disorders and cognitive decline, such as amnestic mild cognitive impairment (aMCI). The objective of this study was to examine the relationship between aMCI and plasma VEGF biomarkers among older PWH. Data were collected at a university-based research center from 2011 to 2013. Participants were 67 antiretroviral therapy-treated, virally suppressed PWH. Participants completed comprehensive neurobehavioral and neuromedical evaluations. aMCI status was determined using adapted Jak/Bondi criteria, classifying participants as aMCI + if their performance was > 1 SD below the normative mean on at least two of four memory assessments. VEGF family plasma biomarkers (i.e., VEGF, VEGF-C, VEGF-D, and PIGF) were measured by immunoassay. Logistic regression models were conducted to determine whether VEGF biomarkers were associated with aMCI status. Participants were mostly non-Hispanic white (79%) men (85%) with a mean age of 57.7 years. Eighteen (26.9%) participants met criteria for aMCI. Among potential covariates, only antidepressant drug use differed by aMCI status, and was included as a covariate. VEGF-D was significantly lower in the aMCI + group compared to the aMCI − group. No other VEGF levels (VEGF, VEGF-C, PIGF) differed by aMCI classification (*p*s > .05). In a sample of antiretroviral therapy-treated, virally suppressed PWH, lower levels of VEGF-D were associated with aMCI status. Longitudinal analyses in a larger and more diverse sample are needed to support VEGF-D as a putative biological marker of aMCI in HIV.

Nearly half of people with HIV (PWH) are aged 50 and older (National Institute on Aging 2020), and the population of older PWH is expected to increase given the widespread use of effective antiretroviral therapy (ART; Centers for Disease Control and Prevention 2016). Older age is the greatest risk factor for developing Alzheimer’s disease (AD) and other age-related neurodegenerative diseases (Guerreiro and Bras 2015). There is a body of literature suggesting probable accelerated, accentuated, and premature aging among PWH (Aung et al. 2020; Pathai et al. 2014; Sheppard et al. 2017). In addition, there are many psychological (e.g., chronic stress, depression, and anxiety; Brandt et al. 2017; Thompson et al. 1996) and biological risk factors (e.g., chronic inflammation, hyperlipidemia, and hypertension; Barbaro 2002) that may make PWH particularly susceptible to age-related neurodegenerative disorders. Thus, as PWH continue to live to older ages, it is possible that PWH are at higher risk for age-related cognitive decline than age-matched HIV-negative (HIV −) persons.

Mild cognitive impairment (MCI) is generally viewed as the intermediate stage between healthy cognitive aging and dementia, and has been identified in individuals before the age of 60 (Kremen et al. 2014). MCI is a mild but noticeable decline in cognition that is steeper than anticipated for an individual’s age but does not yet impair functioning (Gauthier et al. 2006). One proposed MCI typology classifies individuals by impairment type (i.e., amnestic versus non-amnestic; Petersen et al. 2013, 2014). Evidence suggests that amnestic MCI (aMCI; i.e., mild memory deficits regardless of other cognitive domain performance; Kremen et al. 2014) is more closely associated with a higher risk of progression to AD dementia than non-amnestic MCI (Bradfield et al. 2018; Damian et al. 2013; Whitwell et al. 2008). Though the incidence of aMCI and subsequent trajectories among PWH has been understudied, this topic is of increasing public health interest as the HIV population continues to age.

Elevated levels of neuroprotective biomarkers (i.e., brain-derived neurotrophic factor and insulin-like growth factor) have been associated with better cognition among older adults (Bharti et al. 2016; Paulsen et al. 2020; Suh et al. 2015). Other protective factors have been less well studied given the field’s primary focus on neurodegenerative biomarkers. The vascular endothelial growth factor (VEGF) family members have neurotrophic, neuroprotective, and angiogenic properties following brain injury and may have a role in the inhibition of apoptosis (Carmeliet and Ruiz de Almodovar 2013; Gora-Kupilas and Josko 2005; Nag et al. 2019). Higher VEGF levels have been associated with optimal brain aging (i.e., higher hippocampal volume and less hippocampal atrophy/cognitive decline over time; Hohman et al. 2015), particularly among individuals showing early hallmarks of the AD cascade (e.g., elevated levels of tau), as well as better memory and language performance among individuals already diagnosed with AD (Alvarez et al. 2018). Lower expression of VEGF family members has been associated with higher Clinical Dementia Rating scores among individuals diagnosed with AD and aMCI, with higher ratings being indicative of probable dementia (Huang et al. 2013). Further, Provias and Jeynes (2014) found brains from pathologically confirmed AD cases to have significantly less superior temporal, hippocampal, and brainstem capillary expression of VEGF than brains from healthy normal autopsy cases. Thus, levels of VEGF signaling may represent biomarkers associated with protection from aMCI and AD dementia. Although VEGF has been associated with HAND in the context of HIV disease (Kallianpur et al. 2019), it is unknown whether VEGF levels differ across older PWH with and without aMCI.

Few researchers have examined the individual contributions of each VEGF family member to cognitive function. Each VEGF member binds differently to VEGF receptors and is responsible for a unique biological response. For example, VEGF-A is most strongly associated with angio-genesis, VEGF-C and VEGF-D are most strongly associated with lymphangiogenesis, and PIGF is most strongly associated with inflammatory cell recruitment (Rapisarda and Melillo 2012). Most studies of the VEGF family and human neurodegenerative diseases have focused on VEGF-A, often referred to as VEGF. These studies indicate that VEGF-A may play a role in vasculature maintenance and may be neuroprotective against age-related cognitive decline (Garcia et al. 2014; Hohman et al. 2015; Huang et al. 2013; Thomas and Eichmann 2013). Examining other members of the VEGF family may be useful in furthering our understanding of the biological pathways involved in cognitive function.

Though VEGF family members have been implicated in brain aging and AD dementia, it is unknown whether they show alterations earlier in the AD trajectory, i.e., at the aMCI stage. Many studies on VEGF expression have been conducted in vitro and in animal models, with fewer studies focused on the relationship between VEGF family expression and clinical outcomes (Mauceri et al. 2011). Of studies conducted among clinical populations (e.g., AD, cancer; Alvarez et al. 2018; Hohman et al. 2015; Huang et al. 2013; Juttner et al. 2006), none have examined individual VEGF family members in relation to cognitive outcomes among PWH. Yet, this topic is of great importance as protective biomarkers may provide mechanistic insight into the biology of cognitive decline. This study was conducted to examine if biomarkers of the VEGF family are associated with aMCI among older PWH, a group that has been understudied in the VEGF literature and who may be particularly vulnerable to aMCI. We hypothesized that lower levels of biomarkers in the VEGF family would be associated with increased likelihood of aMCI among older PWH.

## Method

### Participants

Participants were 67 PWH who enrolled in the Successfully Aging Seniors with HIV (SASH) study, conducted at the University of California, San Diego (UCSD) HIV Neurobehavioral Research Program. The study was approved by the UCSD Human Research Protections Program, and all participants provided written informed consent. Participants were San Diego community-dwelling adults who were older than 50 years, had HIV infection, and were taking ART with an undetectable plasma viral load (< 50 copies/mL). Participants were excluded if they had a diagnosis of a psychotic disorder or a neurological condition known to adversely affect cognitive function (e.g., stroke).

### Neurocognitive assessment

Participants were administered a neurocognitive battery spanning seven domains commonly affected by HIV: verbal fluency, abstraction/executive functioning, speed of information processing, visual and verbal learning, visual and verbal delayed recall, attention/working memory, and complex motor skills. Raw neurocognitive test scores were converted into demographically adjusted *T* scores to control for the effects of age (normal aging), education, gender, and race/ethnicity (Cherner et al. 2007; Heaton et al. 2004, 2002; Norman et al. 2011). Demographically adjusted *T* scores were used to assign algorithm-derived clinical ratings [ranging from 1 (above average) to 9 (severe impairment)] for each of the seven neurocognitive domains, which were then averaged to derive a global clinical rating (Woods et al. 2004). A global clinical rating of ≥ 5 is indicative of neurocognitive impairment. Consistent with Frascati criteria for HIV-associated neurocognitive disorder (HAND) and the large multi-site CNS HIV Antiretroviral Therapy Effects Research (CHARTER) study (Antinori et al. 2007; Heaton et al. 2010, 2011), at least two domains must be in the impaired range to receive a HAND diagnosis.

### aMCI criteria

Participants were classified into aMCI + or aMCI − groups using adapted Jak/Bondi criteria (Rubin et al. 2019). Jak/Bondi criteria are actuarial diagnostic methods used to classify aMCI in older adults (Bondi et al. 2014; Jak et al. 2009). aMCI classification by Jak/Bondi criteria has been shown to yield greater conversion to AD and have stronger relationships with AD biomarkers than conventional aMCI criteria that rely on rating scales, global cognitive screens, subjective cognitive complaints, and impairment on a single cognitive test (Bondi et al. 2014). Typical Jak/Bondi criteria for aMCI diagnosis require performance > 1 SD below the demographically corrected mean on at least two tests of memory, based on several studies suggesting that a cutpoint for impairment of − 1 SD below normative means provides an optimal balance of sensitivity and specificity (Busse et al. 2006; Jak et al. 2009; Taylor and Heaton 2001). Consistent with a previous study defining aMCI risk among PWH, adapted criteria were used in order to better identify aMCI amid a background of HAND. The adapted criteria requires impairment in at least one recognition memory test given recognition deficits are typical in aMCI but not in HAND (Rubin et al. 2019; Sundermann et al. 2020). Participants were classified as aMCI + if they were impaired on at least two of four tests of memory over a delay period [i.e., Hopkins Verbal Learning Test-Revised (HVLT-R) Recall and Recognition (Brandt and Benedict 2001) and Brief Visuospatial Memory Test-Revised (BVMT-R) Recall and Recognition (Benedict 1997)] with at least one impaired test being either HVLT-R Recognition or BVMT-R Recognition. These adapted Jak/Bondi criteria have been shown to be associated with AD pathology (i.e., amyloid-β_42_ plaque deposition) among autopsy cases of older PWH (Sundermann et al. 2020).

### VEGF biomarkers

Biomarker assays were measured by immunoassay in duplicate in ethylenediaminetetraacetic acid (EDTA)-treated plasma derived from peripheral blood samples collected by routine phlebotomy. The commercial immunoassay supplier was Meso Scale Discovery [Angiogenesis Panel 1 (human): vascular endothelial growth factor-A (VEGF/VEGF-A), vascular endothelial growth factor-C (VEGF-C), vascular endothelial growth factor-D (VEGF-D), and placental growth factor (PIGF); Rockville, MD]. Measurements were repeated if the coefficient of variation was greater than 20% or if the concentration was greater than four standard deviations from the group mean.

### Covariates

Considered covariates included demographic data (e.g., age, education, sex, race/ethnicity), HIV characteristics (e.g., estimated duration of HIV disease, AIDS diagnosis, current and nadir CD4 + *T* cell counts), psychiatric data (e.g., lifetime or current major depressive disorder and current antidepressant use at time of study visit), and medical data (e.g., comorbidities and anthropometric measurements). Given our small sample size, we applied a liberal alpha of 0.10 for identification of covariates associated with aMCI to include in our multinomial logistic regression model.

### Statistical analyses

The distributions of residuals were visually inspected when performing statistical tests to determine whether the use of raw or log-transformed values of plasma VEGF family biomarkers was appropriate to meet statistical assumptions of parametric tests. Based on these inspections, raw values of plasma VEGF family biomarkers were deemed appropriate for use in statistical analyses. Comparisons of demographic, medical comorbidities and anthropometric measurement, medication use, psychiatric characteristics/diagnoses, and HIV disease characteristics between the aMCI + and aMCI − groups were performed using two-tailed *t* tests for continuous variables or Pearson’s chi-squared test for nominal variables. When the assumption of equal variances was not met, Welch’s *t* test was used. Hedge’s *g* statistic for continuous variables and odds ratios for binary variables were used to generate effect sizes for group comparisons.

Pearson’s correlation coefficient was calculated between each VEGF family member for both aMCI groups and the entire sample. This was done to examine which members might be similar to one another, and if similar members relate to aMCI status.

VEGF biomarkers were compared to aMCI by logistic regression modeling, which was performed with and without statistical adjustment for covariates. Covariates were variables in Table 1 that had univariable associations with aMCI status at a critical *α* = 0.10. Only one covariate (antidepressant use) met this criterion and was included in a multinomial logistic regression model. All statistical tests were performed with JMP ® Pro 14.0.0 (Copyright © 2018 SAS Institute, Inc.).

## Results

### Participants

Table 1 presents demographic and clinical characteristic by aMCI status. Of the 67 participants, aMCI groups did not differ in demographic characteristics or HIV characteristics (*p*s > 0.05).

The aMCI + group (85.7%) was more likely than the aMCI − group (20.0%) to be neurocognitively impaired based on global clinical ratings (OR = 24.0, *p* < 0.001). All participants in the aMCI + group also met criteria for HAND (100.0%) while 38.8% of the aMCI − group met criteria for HAND (*p* < 0.001).

**Fig. 1:**
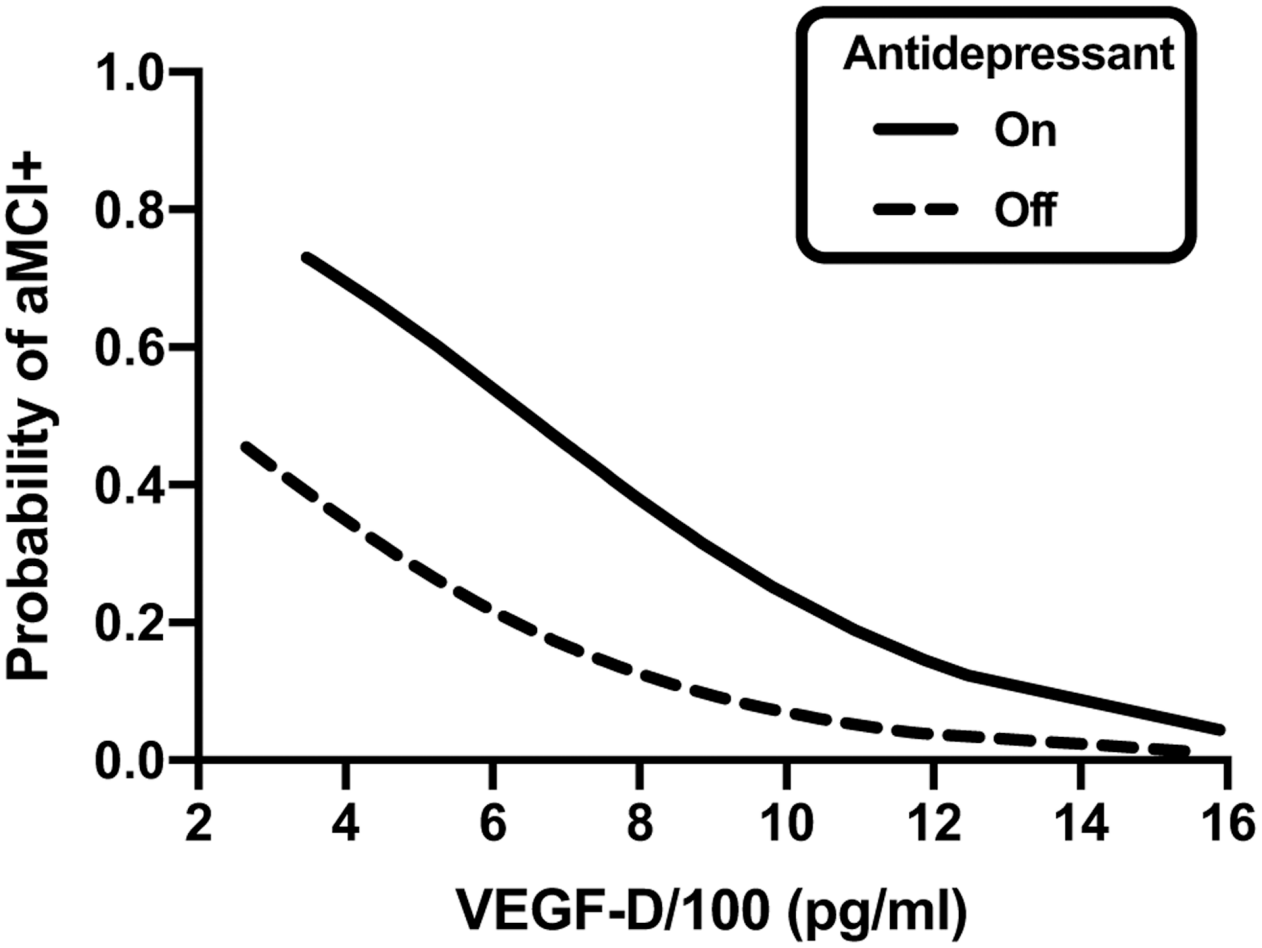
Results from multinomial logistic regression model on aMCI status showing that higher VEGF-D levels (OR = .72 per 100-unit increase in VEGF-D levels, *p* < .01) and antidepressant use (OR = 4.3, *p* < .02) were both associated with higher rates of aMCI (OR = 3.56, *p* = .02)

Of the covariates screened, only antidepressant use was significantly associated with aMCI status (OR = 3.56, *p* = 0.02; Fig. 1). Within the aMCI + group, 61% were on antidepressants at the current visit compared to 31% of the aMCI − group. To explore this association further, we examined whether antidepressant class [i.e., atypical, selective serotonin reuptake inhibition (SSRI), serotonin-norepinephrine reuptake inhibitor (SNRI), and tricyclic] or specific antidepressant (e.g., sertraline) were statistically associated with aMCI status (Table 4). SSRI use approached significance by aMCI status (OR = 4.40, *p* = 0.06), with 33.3% of the aMCI + group and 10.2% of the aMCI − group on an SSRI. No other antidepressant class or medication differed by aMCI status.

### Correlations among VEGF family biomarkers

Table 2 displays a correlation matrix of associations among VEGF family biomarkers within the entire sample, and within each aMCI group separately. Across aMCI groups, VEGF-A was strongly and significantly associated with VEGF-C (*p* < 0.001) and moderately associated with VEGF-D (*p* < 0.05). These associations were also statistically significant when examined within the aMCI − group only (*p*s < 0.05). We found a statistically significant association between VEGF-A and VEGF-C when examined within the aMCI + group only (*p* < 0.05) but no significant association between VEGF-A and VEGF-D (*p* > 0.05). The association between VEGF-A and PIGF was not statistically significant in the overall sample (*p* > 0.05) but was strong and significant when examined in the aMCI + group (*p* < 0.05). None of the other VEGF family biomarkers were significantly associated with each other in the overall sample or when examined within the aMCI groups (*p*s > 0.05).

### The association between VEGF family biomarker levels and aMCI status

Table 3 presents mean levels of VEGF family biomarkers by aMCI status. In unadjusted analyses, the aMCI + group had lower VEGF-D levels compared to the aMCI − group (Hedge’s *g* = −0.74, *p* = 0.01). No other VEGF family biomarker (VEGF, VEGF-C, PIGF) was associated with aMCI status (*ps* > 0.05). When examined in a multinomial logistic regression model controlling for antidepressant use, the association between VEGF-D levels and aMCI status remained statistically significant (model *p* = 0.002). Lower levels of VEGF-D (OR = 0.72 per 100-unit increase in VEGF-D levels, *p* < 0.01) and antidepressant use (OR = 4.3, *p* < 0.02) were associated with higher odds of meeting criteria for aMCI (Fig. 1).

### Post hoc analyses

To disentangle the association between antidepressant use and higher odds of meeting criteria for aMCI status, we conducted a series of post hoc analyses. First, we examined whether antidepressant use was associated with self-reported cognitive symptoms, such as concentration difficulties. Concentration difficulties are commonly assessed by standard depression symptom inventories, such as the Beck Depression Inventory-II (BDI-II; Beck et al. 1996). Self-reported concentration difficulties, as measured by a single item on the BDI-II (“*I find I can’t concentrate on anything*”), were associated with increased odds of being on an antidepressant (OR = 2.83, *p* = 0.05). Next, we examined whether the association between aMCI status and antidepressant use was influenced by the inclusion of antidepressants that may be prescribed to treat sleep problems (i.e., trazodone, mirtazipine, and tricyclics). We therefore created an antidepressant use variable that excluded trazodone, mirtazipine, and tricyclics. This new antidepressant variable was associated with higher odds of meeting criteria for aMCI (OR = 4.32, *p* = 0.01). Lastly, we examined the association between antidepressants prescribed for sleep (i.e., trazodone, mirtazipine, and tricyclics) and aMCI status. We therefore created a sleep medication variable that included trazadone, mirtazipine, and tricyclics; this variable was not associated with aMCI status (OR = 0.30, *p* = 0.30).

## Discussion

As the PWH continue to age, identification of biomarkers that can identify those who may be on an aMCI/AD trajectory is an important unmet need. In this cross-sectional study of older PWH with undetectable viral load, approximately 30% of participants met adapted Jak/Bondi criteria for aMCI. Consistent with our hypothesis and the current cognitive aging literature, lower levels of VEGF-D, an angiogenic protein with neuroprotective effects, was associated with higher odds of meeting aMCI criteria. No other VEGF marker was significantly related to aMCI status. Correlations across VEGF members differed in strength and significance, which may be explained by the processes each member is primarily responsible for, resulting in different associations within the VEGF family. Of the variables screened as potential covariates, only antidepressant use was associated with aMCI classification. It should be noted, however, that the groups did not differ with respect to current or lifetime prevalence of major depressive disorder, or current self-reported depressed mood.

VEGF previously has been implicated in neurogenesis and found to be protective of cognitive functioning (Cao et al. 2004). In a study involving epilepsy-induced rats, VEGF contributed to early neurogenesis and alleviated long-term cognitive deficits (Han et al. 2017). Hippocampal neurogenesis, in particular, appears to positively influence memory and to be protective against cognitive decline (Becker and Wojtowicz 2007; Garcia et al. 2014; Jin et al. 2002; Provias and Jeynes 2014). Results from other animal studies have indicated that genetically increasing hippocampal VEGF resulted in a twofold increase of neurogenesis and improved cognition (During and Cao 2006). Higher expression of VEGF appears necessary to reap the benefits of physical activity on hippocampal neurogenesis (Fabel et al. 2003). Although the present study did not directly examine expression of VEGF in the brain, our finding that VEGF-D concentration in blood was lower in PWH who met criteria for aMCI may motivate future researchers to examine these associations using region-specific levels of VEGF expression in studies involving autopsy cases of older PWH. Ultimately, our study extended this line of research on cognition by examining aMCI as an outcome of VEGF expression.

The body of literature examining the association between VEGF and AD is growing (Alvarez et al. 2018). Among AD transgenic mice, VEGF significantly prevented healthy vascular cell death caused by beta-amyloid buildup (the protein responsible for the formation of Alzheimer’s plaques) and restored memory behavior (Religa et al. 2013). Hohman et al. (2015) suggest that the neuroprotective effects of VEGF are strongest in the presence of AD-related biomarkers and that VEGF may be particularly beneficial in individuals exhibiting early signs of AD. The cognitive profile of aMCI overlaps to some degree with HAND, as indicated by our findings that PWH meeting criteria for aMCI also met criteria for HAND. Thus, to bolster our understanding of whether or not VEGF levels may be an early marker of AD, additional studies that examine VEGF in the context of a pre-clinical stage such as aMCI, such as our study, are needed.

Few studies have examined the neuroprotective properties of VEGF within the aMCI literature. In one study that examined aMCI, a dose-dependent effect of VEGF-A was found in the AD trajectory, such that VEGF levels in participants with aMCI were significantly higher than participants with AD dementia and significantly lower than in participants classified as healthy controls (Huang et al. 2013). No other VEGF family member was examined. These findings suggest that angiogenic markers such as VEGF might longitudinally vary as a function of cognitive impairment severity. By examining individuals diagnosed with aMCI, a potentially transitional stage between normal cognitive aging and AD dementia, our study builds on the clinical relevance of VEGF and lends support to the growing line of research on the higher expression of VEGF family members being a protective factor against cognitive decline.

Research comparing each VEGF family member to cognitive performance is sparse. Recent animal model studies have suggested VEGF-D to be uniquely associated with hippocampal neurogenesis, dendritic maintenance, and cognitive functioning (Mauceri et al. 2011; Stacker and Achen 2018). The link between VEGF-D and hippocampal neurogenesis may be one reason why VEGF-D showed the strongest relationship of the VEGF family markers with aMCI status in this current study. Our study is among the first to examine the VEGF family in relation to aMCI and the first to examine these relationships by individual biomarkers in the VEGF family among PWH. Though our VEGF-D-specific finding requires further verification in larger studies, it lends support that this VEGF subtype may be a biomarker of aMCI among PWH.

We found that the only considered covariate that differed by aMCI status was antidepressant use (i.e., tricyclic antidepressants [TCA], monoamine oxidase inhibitor [MAOI)], SSRI, and/or SNRI). Specifically, a greater proportion of the aMCI + group was currently taking antidepressants, particularly SSRIs, as compared to the aMCI − group. However, lifetime and current major depressive disorder diagnoses, as well as current depression symptoms (i.e., BDI-II score), did not significantly differ between the two aMCI groups. There are many possible reasons for higher prevalence of antidepressant use among the aMCI + group. SSRIs are prescribed for treatment of depression as well as other mental health conditions (e.g., anxiety), which were not assessed in this study. The literature on the relationship between SSRIs and aMCI/AD is somewhat mixed, with some studies finding that SSRIs may be neuroprotective and delay progression of AD (Bartels et al. 2017) whereas others find that SSRI use is associated with increased risk of dementia (Kessing et al. 2009). However, the mental health conditions that SSRIs treat (e.g., late-life depression; Steenland et al. 2012) and the negative effects SSRIs may have on sleep (Ju et al. 2014) are associated with increased risk of aMCI/AD in the general population. It is also possible that the higher prevalence of antidepressant use among the aMCI + group is due to the possibility that clinicians treating these patients (or the patients themselves) may have interpreted the patient’s reports of concentration difficulties and/or forgetfulness as a sign of depression. Consequently, these patients may be prescribed an antidepressant. Although we found antidepressant use to be higher among aMCI + individuals, future studies should examine the potential underlying conditions for which antidepressants are prescribed among PWH.

The study has several limitations. First, the sample size of this study was relatively small, only including participants on ART with an undetectable viral load, and study participants were primarily non-Hispanic white men. Therefore, these findings may not be generalizable to all older PWH. Second, because this study was cross-sectional in nature, causality should not be assumed. Longitudinal studies that examine VEGF along with inflammatory markers, AD biomarkers, and hippocampal atrophy could help better elucidate how VEGF-D is related to risk of aMCI among older PWH. Third, most published manuscripts measure VEGF-A without measuring other markers of the VEGF family, making comparisons to our study difficult. Our findings support that measuring markers of the VEGF family may provide valuable insights. The difference in VEGF-D values by aMCI status was statistically significant but small (median 2.8 in the aMCI + group and median 2.9 in the aMCI − group). Additional research is needed to explore clinically meaningful differences in VEGF values. Fourth, it is well documented that VEGF is elevated in the presence of cerebrovascular disease (Matsuo et al. 2013; Shim and Madsen 2018; Zheng et al. 2017). The present study did not examine the prevalence of cerebrovascular disease across our sample, which may increase the likelihood of receiving an aMCI diagnosis, and more specifically, may explain the relationship between VEGF and aMCI. Relatedly, we did not systematically collect detailed information from participants about the management of medical comorbidities (e.g., type of medication prescribed for diabetes management). The degree to which medical comorbidities are successfully treated may influence biomarker levels and/or presence of aMCI. Last, this study did not include an HIV-negative comparison group. Nevertheless, this study represents an important first step in examining peripheral biomarker correlates of aMCI among older PWH.

In conclusion, we examined the relationship between four VEGF family members and aMCI among PWH. Our study found that VEGF-D was specifically was associated with aMCI status among PWH whereas other VEGF biomarkers were not. These findings underscore the importance of examining the individual VEGF family members in relation to cognitive outcomes. As PWH continue to live longer lives, identifying novel biomarkers that can help to identify persons who may or may not be on a neurodegenerative AD trajectory will allow for early intervention and treatment, and ultimately promote longevity and quality of life for PWH.

## Figures and Tables

**Table 1:** Participant characteristics by aMCI status (*n* = 67)

Variable	aMCI − (*n* = 49)	aMCI + (*n* = 18)	*p* value	Effect size
**Descriptive demographics**				
Age, mean (SD)	57.7 (6.7)	57.8 (4.9)	.97	*g* = .02
Education, mean (SD)	14.2 (2.7)	15.1 (2.4)	.20	*g* = .34
Male, *n* (%)	41 (84%)	16 (89%)	.59	OR = 1.56
Non-Hispanic white, *n* (%)	37 (76%)	16 (89%)	.39	OR = 2.59
**Medical comorbidities and anthropometric measurement**				
Hyperlipidemia, *n* (%)	26 (57%)	11 (61%)	.74	OR = 1.21
Ever smoker, *n* (%)	18 (39%)	7 (39%)	.99	OR = 0.99
Hypertension, *n* (%)	18 (39%)	8 (44%)	.70	OR = 1.24
Current smoker, *n* (%)	16 (35%)	6 (33%)	.91	OR = 0.94
Diabetes, *n* (%)	13 (28%)	4 (22%)	.62	OR = 0.73
Hepatitis C virus, *n* (%)	9 (20%)	5 (28%)	.48	OR = 1.58
Body mass index, mean (SD) ^a^	26.3 (5.2)	28.5 (6.3)	.15	*g* = .39
Pulse pressure	58.8 (18.3)	58.3 (19.4)	.92	
**Current medications**				
Lipid-lowering drug, *n* (%)	18 (37%)	8 (44%)	.57	OR = 1.38
NSAID, *n* (%)	15 (31%)	5 (28%)	.82	OR = 0.87
Antihypertensive, *n* (%)	12 (24%)	8 (44%)	.12	OR = 2.47
Antidepressant, *n* (%)	15 (31%)	11 (61%)	.02	OR = 3.56
**Psychiatric characteristics/diagnoses**				
BDI-II total, median [IQR]	8.0 [2.0, 16.0]	8.5 [1.8, 15.8]	.97	
Current MDD, *n* (%)^a^	5 (10%)	3 (17%)	.50	OR = 1.72
LT MDD, *n* (%)^b^	27 (55%)	11 (61%)	.66	OR = 1.28
LT alcohol use disorder, *n* (%)^b^	23 (47%)	8 (44%)	.86	OR = 0.90
LT cannabis use disorder, *n* (%)^b^	13 (27%)	5 (28%)	.92	OR = 1.07
LT meth use disorder, *n* (%)^b^	4 (22%)	15 (31%)	.49	OR = 0.65
**HIV disease characteristics**				
Est. duration of HIV disease, (years) median [IQR]^a^	20 [11.9, 25.1]	16.2 [10.9, 25.3]	.82	
AIDS, *n* (%)^b^	32 (65%)	11 (61%)	.75	OR = 0.83
Current CD4, median [IQR]	597 [363, 775]	663 [566, 1,049]	.11	
Nadir CD4, median [IQR]^a^	165 [21, 300]	140 [42, 303]	.75	

*BDI-II* Beck Depression Inventory-II, *LT* lifetime, *MDD* major depressive disorder, *NSAID* nonsteroidal anti-inflammatory drug

**Table 2:** Correlation matrix among VEGF family biomarkers

	VEGF-A	VEGF-C	VEGF-D
**VEGF-C**			
All (*N* = 67)	.73***		
aMCI − (*n* = 49)	.78***		
aMCI + (*n* = 18)	.57*		
**VEGF-D**			
All (*N* = 67)	.29*	.10	
aMCI − (*n* = 49)	.33*	.18	
aMCI + (*n* = 18)	.40	.12	
**PIGF**			
All (*N* = 67)	.24	.20	.14
aMCI − (*n* = 49)	.16	.16	.19
aMCI + (*n* = 18)	.55*	.25	.30

* *p* < .05,

** *p* < .01,

*** *p* < .001

**Table 3:** Median levels of VEGF family biomarkers by aMCI status

Biomarker	aMCI + (*n* = 18)	aMCI − (*n* = 49)	*p* value	Effect size
VEGF-A (pg/dL)	2.2 [2, 2.3]	2 [1.9, 2.3]	0.43	*g* = .05
VEGF-C (pg/mL)	2.1 [1.8, 2.3]	1.9 [1.8, 2.2]	0.20	*g* = .60
VEGF-D (pg/mL)	2.8 [2.6, 2.9]	2.9 [2.8, 3]	**0.01**	*g* = −.74
PIGF (pg/mL)	1.5 [1.4, 1.5]	1.4 [1.4, 1.5]	0.15	*g* = .39

**Table 4:** Antidepressant use by aMCI status (*n* = 67)

Variable	aMCI − (*n* = 49)	aMCI + (*n* = 18)	*p* value	Effect size
On antidepressant, *n* (%)	15 (30.6%)	11 (61.1%)	.02	OR = 3.56 (1.16, 10.98)
On atypical, *n* (%)	9 (18.4%)	5 (27.8%)	.50^a^	OR = 1.71 (0.49, 6.02)
On bupropion HCL, *n* (%)	4 (8.2%)	3 (16.7%)	.38^a^	OR = 2.25 (0.45, 11.22)
On trazodone HCL, *n* (%)	4 (8.2%)	3 (16.7%)	.38^a^	OR = 2.25 (0.45, 11.22)
On mirtazapine, *n* (%)	2 (4.1%)	2 (11.1%)	.29^a^	OR = 2.94 (0.38, 22.60)
On SSRI, *n* (%)	5 (10.2%)	6 (33.3%)	.06^a^	OR = 4.40 (1.14, 16.93)
On escitalopram oxalate, *n* (%)	3 (6.1%)	1 (5.6%)	1.00^a^	OR = 0.90 (0.09, 9.28)
On fluoxetine, *n* (%)	2 (4.1%)	1 (5.6%)	1.00^a^	OR = 1.38 (0.12, 16.24)
On sertraline HCL, *n* (%)	0 (0.0%)	2 (11.1%)	.07^a^	
On paroxetine, *n* (%)	0 (0.0%)	1 (5.6%)	.27^a^	
On citalopram HBR, *n* (%)	0 (0.0%)	1 (5.6%)	.27^a^	
On SNRI, *n* (%)	4 (8.2%)	1 (5.6%)	1.00^a^	OR = 0.66 (0.07, 6.35)
On venlafaxine HCL, *n* (%)	3 (6.1%)	1 (5.6%)	1.00^a^	OR = 0.90 (0.09, 9.28)
On duloxetine HCL, *n* (%)	1 (2.0%)	0 (0.0%)	1.00^a^	
On tricyclics, *n* (%)	3 (6.1%)	1 (5.6%)	1.00^a^	OR = 0.90 (0.09, 9.28)
On amitriptyline HCL, *n* (%)	2 (4.1%)	1 (5.6%)	1.00^a^	OR = 1.38 (0.12, 16.24)
On desipramine, *n* (%)	1 (2.0%)	0 (0.0%)	1.00^a^	

a *p* value corresponds to Fisher’s exact test
